# Adverse obstetric outcomes in pregnant women with uterine fibroids in China: A multicenter survey involving 112,403 deliveries

**DOI:** 10.1371/journal.pone.0187821

**Published:** 2017-11-14

**Authors:** Rong Zhao, Xin Wang, Liying Zou, Guanghui Li, Yi Chen, Changdong Li, Weiyuan Zhang

**Affiliations:** Department of Obstetric medicine, Beijing Obstetrics and Gynecology Hospital, Capital Medical University, Beijing, People’s Republic of China; Stellenbosch University, SOUTH AFRICA

## Abstract

**Objective:**

To estimate the association between uterine fibroids and adverse obstetric outcomes.

**Methods:**

This was a retrospective cross-sectional study of 112,403 deliveries from 14 provinces and 39 different hospitals in 2011 in mainland China. We compared pregnancy outcomes in women with and without uterine fibroids who underwent detailed second trimester obstetric ultrasonography during 18 to 22 weeks. Obstetric outcomes include cesarean delivery, breech presentation, preterm delivery, placenta previa, placental abruption, premature rupture of membranes and neonatal birthweight. Univariate analyses and multivariate logistic regression analyses were performed.

**Results:**

Of 112,403 women who underwent routine obstetric survey, 3,012 (2.68%) women were identified with at least 1 fibroid. By univariate and multivariate analyses, the presence of uterine fibroids was significantly associated with cesarean delivery (Adjusted odds radio [AOR] 1.8, 95% confidence interval [CI] 1.7–2.0), breech presentation (AOR 1.3, 95% CI 1.2–1.5) and postpartum hemorrhage (AOR 1.2, 95% CI 1.1–1.4). The size of uterine fibroids and location in uterus had important effect on the mode of delivery. The rates of PPH were significantly higher with increasing size of the uterine fibroid (P<0.001). And the location of fibroid (intramural, submucosal or subserosal) also have a statistically significant impact on the risk of PPH (5.6% [subserosal] vs 4.7% [submucosal] vs 8.6% [intramural]).

**Conclusion:**

Pregnant women with uterine fibroids are at increased risk for cesarean delivery, breech presentation and postpartum hemorrhage. And different characteristics of uterine fibroids affect obstetric outcomes through different ways. Such detailed information may be useful in risk-stratifying pregnant women with fibroids.

## Introduction

Uterine fibroids are the most common benign tumors of the female reproductive tract [1]. According to the trimester of assessment and the size threshold used, the prevalence of uterine fibroids among pregnant women ranges from less than 1% to 10.7% [2,3]. As more and more women delay child bearing to later in life, the prevalence of uterine fibroids during pregnancy is likely to increase. The incidence of uterine fibroids increases with age [4]. At present, although there are a lot of research about the prevention and treatment of uterine fibroids [5,6,7], the etiopatogenesis of uterine fibroids is still unclear [8,9].

There are conflicting data on the relationship between obstetric outcomes and uterine fibroids, and the mechanism by which fibroids influence obstetric outcomes is unclear. Some studies have shown a relationship between uterine fibroids and pregnancy complications, such as preterm birth, premature rupture of membranes (PROM), fetal malpresentation, placental abruption and intrauterine fetal demise [10,11,12]. In addition, uterine fibroids have been linked to labor dystocia, puerperal infection, operative vaginal delivery, cesarean delivery and postpartum hemorrhage (PPH) [2,13]. In contrast, other studies have reported no increased risks for these adverse obstetric outcomes with uterine fibroids [14]. More recent studies have attempted to clarify these conflicting results by grouping fibroids by size and location [15, 16], but those studies still obtained conflicting results due to their small sample sizes.

The objective of this study was to assess the impact of uterine fibroids on obstetric outcomes, especially the association between different characteristics of uterine fibroid and adverse obstetric outcomes.

## Materials and methods

This was a multi-center, cross-sectional, retrospective survey of women who delivered at 39 hospitals (19 tertiary care hospitals and 20 secondary care hospitals) in 14 provinces in China, from January to December 2011. All participating facilities were chosen from seven territories of China using multistage stratified random sampling.

In the Chinese healthcare system, a tertiary hospital provides the highest level of medical service, and a secondary hospital provides a middle level of care. Primary care hospitals usually only provide low-level care and are not equipped to perform caesarean sections due to the lack of an emergency obstetric or neonatal care unit. Because most inpatients (94.4%) are admitted to tertiary and secondary hospitals, as reported in the China Statistical Yearbook 2013, all the institutions included in the present study have been selected between tertiary and secondary hospitals. In our research, all pregnant women took regular prenatal cares: once a month before 28 weeks, every two weeks between 28–36 weeks, once a week after 36 weeks. We obtained all maternal and fetal data, including maternal characteristics, gestational care, intrapartum care, delivery care, postpartum care, newborn baby care, ultrasonographic tests and laboratory tests through medical records review. The collected data included complications of gestation, mode of delivery and maternal and perinatal outcomes. In our study, alcohol assumption means alcohol use during pregnancy, current smoker means pregnant women using tobacco. Preterm delivery was defined as birth occurring between 28 and 37 weeks of gestation. Gestational diabetes mellitus (GDM) is defined as an abnormal glucose tolerance of varying degree that is detected for the first time during pregnancy. The diagnostic criteria of GDM are blood glucose levels during fasting and at the first hour and second hour after an oral glucose tolerance test (OGTT), with cut-offs of 5.1, 10.0, and 8.5 mmol/L (92, 180, and 153 mg/dl), respectively. Diabetes mellitus (DM) includes pregnant women with pre-existing type 1 or type 2 diabetes. Hypertensive disorder complicating pregnancy (HDCP) includes gestational hypertension, preeclampsia, eclampsia and chronic hypertension complicating pregnancy. Postpartum hemorrhage (PPH) is defined as estimated blood loss of more than 1000ml for cesarean and vaginal delivery. Low birth weight infant (LBW) is defined as birth weight less than 2500g. All pregnant women had a detailed ultrasound examination during 18 to 22 weeks. The detailed data of fibroids in these ultrasound examinations were collected for analysis. All sonographers have been properly trained and licensed in ultrasound. The uterine fibroid group included pregnant women with at least 1 fibroid. The control group included pregnant women with no ultrasonographically detected fibroids. The following clinical characteristics and adverse obstetric outcomes were analyzed: maternal age, gestational age at delivery, gravidity, parity, BMI, smoking status, alcohol assumption, GDM, DM, HDCP, previous preterm birth, cesarean delivery, preterm birth, breech presentation, premature rupture of membranes (PROM), placenta previa, placenta abruption, PPH and LBW. All medical records of pregnant women and their babies were reviewed and data extracted by a trained group of physicians before patients were discharged. All the data were recorded on a paper form, and then entered into computers and uploaded to the network database. Each sub-center of province/municipality/autonomous region sent three properly trained inspectors. They were responsible for training their own data-entry clerks in every sub-center. In every province/municipality/autonomous region, two professionals who underwent the same training were in charge of the initial quality control. A professional staff member performed the second quality control process after the data had been uploaded to the database. This study was approved by the Ethics Committees of medical institutions (Capital Medical University Institutional Review), which involved and conformed to the guidelines of the Helsinki agreement and its amendments. The National Research Ethics Service had previously approved the anonymous use of these data for research purposes, so individual informed consent was not required. We obtained all clinical information by reviewing patient’s clinical medical recording files retrospectively, not by face-to face or direct telephone calls. All participants’ personal information, such as mother’s name, phone number and home address, were eliminated from the survey to ensure patient privacy.

The statistical analysis was performed using SPSS version 22 software. Data are presented as percentages or means ±SD. The prevalence of obstetric complications was compared between pregnant women with fibroids and women without fibroids. Categorical variables were compared between groups using the chi-square or Fisher's exact test. P values were considered to be significant at < 0.05. Univariate analyses were performed separately for the assessment of the association between uterine fibroids and obstetric outcomes. The variables that were significantly associated with the outcome in the bivariate analysis (p < 0.05) were included in the multivariate logistic regression analysis to determine the relationships between uterine fibroids and the risks of different adverse obstetric outcomes, and the results are presented as adjusted ORs with corresponding 95% CIs.

## Results

**Fig 1:**
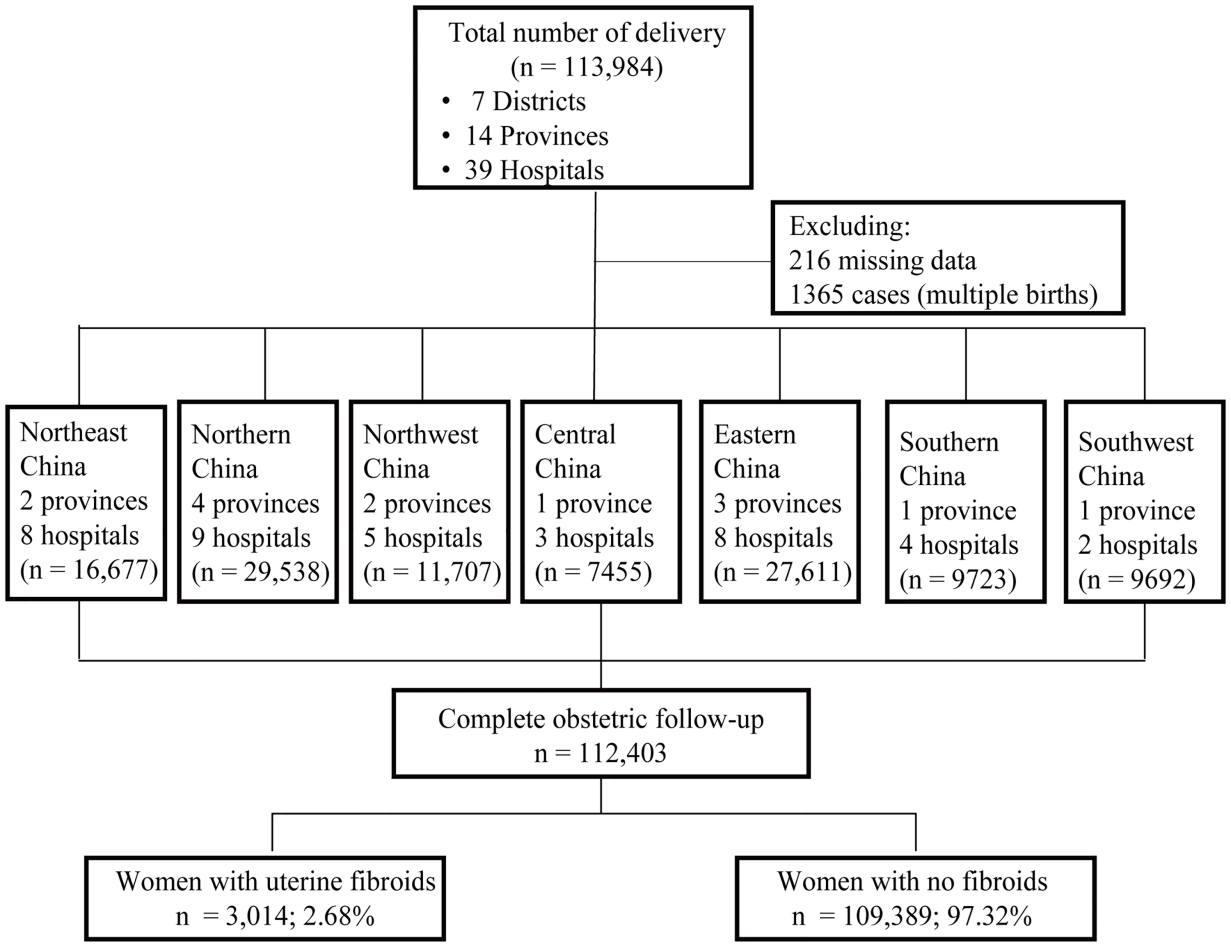
Flow progress chart of study population.

From January 1 2011, to December 31 2011, 112,403 women who delivered in 39 hospitals in China were surveyed for our study. Of all 112,403 women who received routine prenatal care, including ultrasound survey, the prevalence of uterine fibroids was 2.68% (n = 3,012) (Fig 1).

Women with uterine fibroids were older and more likely to have a higher body mass index. Because of family planning and the one-child policy in China, primiparous women are very common in China. In our study, women with high gravidity and low parity were more likely to have uterine fibroids, and both of these differences were significant (p < 0.01). In addition, women with fibroids were more likely to be smokers and drinkers and more likely to have gestational diabetes mellitus (GDM), diabetes mellitus (DM) and hypertensive disorder complicating pregnancy (HDCP) than women without fibroids (Table 1).

**Table 1:** Sociodemographic and clinical characteristics of pregnant women with and without uterine fibroids.

Characteristics	Uterine fibroids (n = 3012)	No fibroids (n = 109,391)	P
**Mean maternal age (y)**	32.0±4.9	27.9±5.2	< 0.01
**Mean Gestational age at delivery (wk)**	38.4±2.3	39.0±2.5	< 0.01
**Gravidity**	1.93±1.2	1.85±1.1	< 0.01
**Parity**	1.15+0.4	1.21±0.5	< 0.01
**Body mass index(kg/m**^**2**^**)**	22.4±3.1	21.6±3.0	< 0.01
**Current smoker**	3.65	2.47	< 0.01
**Alcohol assumption**	1.53	0.87	< 0.01
GDM*	12.4	4.4	< 0.01
DM**	0.3	0.1	< 0.01
**HDCP**^#^	8.4	4.5	< 0.01
**Previous preterm birth**	0.5	0.2	<0.01

Data are means± standard deviation or %.

P for comparison of uterine fibroids group with no fibroids group.

*GDM, gestational diabetes mellitus

**DM, diabetes mellitus

^#^HDCP, hypertensive disorder complicating pregnancy

Among the women with uterine fibroids, 2606 had a uterine fibroid diameter of 5 cm or smaller, and 406 had a uterine fibroid diameter more than 5cm. Additionally, 2210 patients (73.4%) had a single fibroid, whereas 802 patients (26.6%) had multiple fibroids. Fibroids were found to be subserosal in 1948 patients (64.7%), submucosal in 1000 patients (33.2%), and intramural in 64 patient (2.1%).

The cesarean delivery rate for women with fibroids was 85.2% compared to 53.5% for the no fibroid group (P < 0.001). We were able to confirm the association between uterine fibroids and an increased risk for cesarean delivery (OR 2.2, 95%CI 2.1–2.3) even after excluding pregnancies with breech presentation, placenta previa and placental abruption which would by routine standard of care requires a cesarean delivery, and controlling for maternal age, parity, body mass index, hypertensive disorder complicating pregnancy, PROM, neonatal weight, gestational age at delivery determined by ultrasonography (adjusted OR 1.7, 95%CI 1.6–1.9). To evaluate the exact effect of uterine fibroid on cesarean section, a subgroup analysis was performed in which the cesarean section only by maternal request group has been removed. Among the subgroup of women who did cesarean delivery with indications, there were consistently higher rates of cesarean delivery for the uterine fibroids group compared with no fibroids group (OR 2.4, 95%CI 2.3–2.5 and the adjusted OR 1.8, 95%CI 1.7–2.0).

In addition, we found that the presence of fibroids was associated with increased risks of breech presentation (6.9% vs. 3.5%, adjusted OR 1.3, 95% CI 1.2–1.5) and PPH (6.6% vs 3.7%, adjusted OR 1.2, 95%CI 1.1–1.4). The prevalences of placenta previa and PROM were higher in women with fibroids, but the differences were not statistically significant. Increased risks of preterm birth and placental abruption in women with fibroids compared to women without fibroids were not observed even after controlling for potential confounding factors of obstetric outcomes (Table 2).

**Table 2:** Association between uterine fibroids and obstetric and delivery outcomes by multiple logistic regression.

Outcome	Uterine fibroids (n = 3,012)	No fibroids (n = 109,391)	OR	Adjusted OR (only by age)	Adjusted OR	P
Cesarean delivery(all subject)*	85.2	53.5	2.2(2.1–2.3)	2.1(2.0–2.2)	1.7(1.6–1.9)	<0.001
**Cesarean delivery (without only maternal request group)** *	72.7	39.0	2.4(2.3–2.5)	2.2.(2.1–2.3)	1.8(1.7–2.0)	<0.001
Breech presentation**	6.9	3.5	1.4(1.3–1.5)	1.4(1.3–1.5)	1.3(1.2–1.5)	<0.001
Preterm delivery***	9.1	8.2	1.0(0.9–1.1)	1.0(0.9–1.0)	1.0(0.9–1.2)	0.906
**Placenta previa**^#^	1.9	1.2	1.3(1.1–1.4)	1.0(0.9–1.2)	1.1(1.0–1.3)	0.168
**Placental abruption**^##^	0.6	0.5	1.1(0.9–1.4)	1.0(0.8–1.3)	1.0(0.8–1.2)	0.75
**PROM**^###^	18.2	16.2	1.0(0.9–1.1)	1.0(0.9–1.0)	1.0(1.0–1.1)	0.218
**PPH**^&^	6.6	3.7	1.9(1.6–2.2)	1.2(1.1–1.3)	1.2(1.1–1.4)	<0.001
**LBW**^&&^	7.9	7.0	0.9(0.8–1.0)	0.9(0.9–1.0)	1.0(0.9–1.1)	0.966

CI, confidence interval; PROM, premature rupture of membranes, LBW, low birth weight infant.

Data are % unless otherwise specified

* excluding women with breech presentation, placenta previa and placental abruption, and controlling for maternal age, parity, body mass index, hypertensive disorder complicating pregnancy, prom, neonatal weight, gestational age at delivery determined by ultrasonography.

** After controlling for maternal age, parity, body mass index, hypertensive disorder complicating pregnancy, GDM, alcohol use, neonatal weight.

***After controlling for maternal age, parity, body mass index, hypertensive disorder complicating pregnancy, GDM, prom, tobacco use, alcohol use, previous preterm birth.

^#^After controlling for maternal age and gravidity.

^##^ After controlling for maternal age, hypertensive disorder complicating pregnancy, PROM, smoking.

^###^After controlling for maternal age, smoking, alcohol, BMI

^&^After controlling for maternal age, parity, BMI, hypertensive disorder complicating pregnancy, GDM, placenta previa, placental abruption, mode of delivery and neonatal weight.

^&&^After controlling for maternal age, BMI, hypertensive disorder complicating pregnancy, GDM, smoking and alcohol,

Uterine fibroids size was categorized into three groups (<2cm, 2-5cm, and >5cm). The rate of PPH were higher with increasing size of the uterine fibroid, and the difference was significant (P<0.001). The mode of delivery and placenta previa were different during the three groups of fibroid size (P<0.001 and P = 0.034). However, the uterine fibroid size did not affect the PROM, placental abruption, preterm birth (<37 weeks) and preterm birth (<34 weeks) (Table 3).

**Table 3:** Relationship between size of uterine fibroid and obstetric outcomes.

Variable	<2cm (n = 1240)	2-5cm (n = 1366)	>5cm (n = 406)	P
**PROM**	200(16.1%)	221(16.2)	66(16.3%)	0.998
**Placental abruption**	25(2.0%)	25(1.8%)	7(1.7%)	0.908
**Placenta previa**	12(1.0%)	3(0.2%)	4(1.0%)	0.034
**Preterm birth(<37 week)**	111(9.0%)	112(8.2%)	37(9.2%)	0.728
**Preterm birth (< 34 week)**	56(4.5%)	60(4.4%)	16(3.9%)	0.886
**Mode of delivery**				<0.001
** Vaginal delivery**	120(9.7%)	239(17.5%)	63(15.5%)	
** Cesarean delivery (all subject)**	1116(90%)	1110(81.3%)	339(83.5%)	
** Cesarean delivery (without only maternal request group)**	907(88%)	973(79.2)	310(82.2%)	
** Instrument**	4(0.3%)	17(1.2%)	4(1.0%)	
**PPH**	58(4.7%)	101(7.4%)	40(9.9%)	<0.001

We compared the single fibroid group and multiple fibroids group. The mode of delivery is significantly different between women with a single fibroid and patients with multiple fibroids (P < 0.002), while the rate of cesarean section was higher in women with multiple fibroids compared with those with a single fibroid (89% vs 83.8%). Although women with multiple fibroids were more likely to have placenta previa compared with women with a single fibroid (2.6% vs 1.6%), this difference was not significant (P = 0.078). The rate of PPH was slightly higher in the single fibroid group than the multiple fibroids group, but the difference was not significant (6.7% vs 6.2%, P = 0.62) (Table 4).

**Table 4:** Relationship between number of uterine fibroids and obstetric outcomes.

Variable	Single fibroid (n = 2210)	Multiple fibroids (n = 802)	P value
**PROM**	365(16.5%)	122(15.2%)	0.39
**Placental abruption**	13(0.6%)	6(0.7%)	0.624
**Placenta previa**	36(1.6%)	21(2.6%)	0.078
**Preterm birth(<37 week)**	189(8.6%)	71(8.9%)	0.795
**Preterm birth (< 34 week)**	99(4.5%)	33(4.1%)	0.665
**Mode of delivery**			0.002
** Vaginal delivery**	339(15.3%)	83(10.3%)	
** Cesarean delivery**	1851(83.8%)	714(89%)	
** Cesarean delivery (without only maternal request group)**	1557(81.3%)	632(87.8%)	
** Instrument**	20(0.9%)	5(0.6%)	
**PPH**	149(6.7%)	50(6.2%)	0.62

Furthermore, we compared the exact localization of uterine fibroids (intramural, submucosal or subserosal). The location of fibroid (intramural, submucosal or subserosal) have a statistically significant impact on the rates of PPH (5.6% [subserosal] vs 4.7% [submucosal] vs 8.6% [intramural]). There was no difference in PROM or placental abruption between subserosal and intramural fibroids. Although women with submucosal fibroids and intramural fibroids had relatively higher rates of cesarean sections compared with patients with subserosal fibroids, this difference was not significant. There was no significant effect of the location of the fibroids on the rates of preterm delivery (< 37 weeks) and preterm delivery (< 34 weeks) (Table 5).

**Table 5:** Relationship between location of uterine fibroids within the uterus and obstetric outcomes.

Varible	Subserosal (n = 1948)	submucosal (n = 64)	intramural (n = 1000)	P
**PROM**	323(16.6%)	5(7.8%)	159(15.9%)	0.896
**Placental abruption**	36(1.8%)	1(1.6%)	20(2.0%)	0.942
**Placenta previa**	12(0.6%)	0	7(0.7%)	0.243
**Preterm birth(<37 week)**	176(9.1%)	2(3.1%)	82(8.2%)	0.207
**Preterm birth (< 34 week)**	92(4.7%)	2(3.1%)	38(3.8%)	0.452
**Mode of delivery**				0.623
** Vaginal delivery**	285(14.6)	7(10.9%)	130(13.0%)	
** Cesarean delivery**	1646(84.5%)	57(89.1%)	862(86.2%)	
** Cesarean delivery (without only maternal request group)**	1393(82.2%)	52(88.1%)	744(84.4%)	
** Instrument**	17(0.9%)	0	8(0.8%)	
**PPH**	110(5.6%)	3(4.7%)	86(8.6%)	0.008

## Discussion

With the increase in childbearing age, fibroids are becoming more common in pregnant women. The high prevalence of uterine fibroids in the general population makes the effects of uterine fibroids durithe antepartum, intrapartum, and postpartum periods highly remarkable. In our study, 112,403 pregnant women in 39 hospitals in China were surveyed, and we confirmed that the presence of fibroids increased the risk of adverse obstetric outcomes. The prevalence of uterine fibroids in our Chinese study population was 2.68%, which is similar to the prevalence reported in previous studies [10,13,14,17,18].

As described in other studies, the presence of uterine fibroids was associated with advanced maternal age, gravidity, DM, GDM and HDCP [19]. We also found an association between uterine fibroids and BMI and smoking and alcohol assumption.

In our research, we confirmed that uterine fibroids were significantly associated with cesarean delivery in which the data of cesarean section only by maternal request has been removed, even after excluding diagnoses, such as placenta previa and placental abruption. Data from previous studies regarding these outcomes has been conflicting. Sheiner et al [19] reported a nearly 7-fold higher rate of cesarean delivery in women with uterine fibroids than in women without fibroids. Coronado et al [2] described a 6-fold higher rate of cesarean delivery in women with fibroids than in controls. Many studies [10,18, 20,21] have reported that uterine fibroids increased the risk of cesarean delivery. And the results of a study by Michels KA et al [22] supported the relationship between uterine fibroids and an increased risk of cesarean delivery, particularly with larger tumor volumes. However, several studies have also shown no increase in the risk of cesarean delivery in women with fibroids [23,24,25].

We found that the presence of uterine fibroids was associated with an increased risk for breech presentation (6.9% compared with 3.5%, adjusted OR 1.3, 95%CI 1.2–1.5). The relationship remained even after controlling for confounding variables such as maternal age, parity, body mass index, gestational hypertension, GDM, alcohol use and neonatal weight. We evaluated the subgroup of women with fibroids larger than 5cm and found an increased risk for breech presentation among women with large fibroids. Similarly, Qidwai et al describing an increased risk for malpresentation among women with uterine fibroids larger than 10cm compared with women with fibroid less than 10cm [10].

Data from previous studies were inconsistent regarding the association between uterine fibroids and PROM. Stout M J et al [18] reported that women with uterine fibroids had higher rates of PROM than women without fibroids (3.3% vs. 2.4%, adjusted OR 1.3, 95% CI 1.0–1.7). In contrast, other studies have reported that PROM was not associated with uterine fibroids [10], which is consistent with our research. Unlike the results of some previous studies [10,18], our study results showed no association between uterine fibroids and placental abruption or placenta previa.

In this study, we have not only concentrated on important outcomes, such as preterm delivery, mode of delivery, and postpartum hemorrhage, but also have tried to analysis these outcomes with different aspects of fibroids (size, number, location), which thus far has not been clearly elucidated from previous studies.

In the previous studies, the risk of postpartum hysterectomy has been demonstrated to be higher in women with uterine fibroids [21,26], and the risk of PPH are also higher in patients with fibroids [27,28,29], while other studies showed no increase in the rate of postpartum hemorrhage in the women with fibroids [2, 30]. In our research, the presence of uterine fibroids was associated with a significantly increased risk of PPH (OR 1.9, 95%CI 1.6–2.2, P<0.001). The relationship remained even after controlling for confounding variables such as maternal age, parity, BMI, hypertensive disorder complicating pregnancy, GDM, placenta previa, placental abruption, mode of delivery and neonatal weight. The results of our study also show that not only the size but also its location in the uterus influences the risk of postpartum hemorrhage, while the number of fibroids has no effect on the risk of PPH. The association between different features of uterine fibroids and PPH has been discussed rarely in the previous studies and it should be useful for helping identify the risk of PPH in the pregnant women with fibroids.

Our study had some limitations. Because our study population was chosen strictly from secondary and tertiary hospitals, people who live in rural areas and people hospitalized at primary medical institutions were excluded. It is also a limitation of our study for lacking data about the extremely preterm birth (<28 weeks). Furthermore, there may have been some unidentified confounders, although we made great effort to account for all confounding factors.

The major strengths of our study are that it included a large study population and provided important data on maternal obstetric complications and perinatal outcomes in mainland China. In our research, all fibroids were identified during routine prenatal scan, while all of ultrasound data come from second trimester (18–22 weeks), because some uterine fibroids may be covered by the fetal body during third trimester. In this large retrospective study, more details about how obstetric outcome is influenced by different characteristics of uterine fibroids have been showed. The rate of PPH was positively correlated with the increasing size of uterine fibroid, and the location of fibroids also influence the risk of PPH. Mode of delivery was found to be influenced by the size and number of uterine fibroid. Such information may be useful in risk-stratifying pregnant women with fibroids.

Approximately 2.68% of pregnant women in mainland China have been reported to have uterine fibroids, which are related to increased risks of cesarean delivery, breech presentation and PPH. And more details have been discussed about obstetric outcome and different characteristics of uterine fibroids. We hope our study provides clinically useful data for helping clinicians counseling and treating women with uterine fibroids during pregnancy.

## References

[1] Levy BS: Modern management of uterine fibroids. Acta obstetricia et gynecologica Scandinavica 2008, 87(8):812–823. doi: 10.1080/00016340802146912 18607823 10.1080/00016340802146912

[2] Coronado GD, Marshall LM, Schwartz SM: Complications in pregnancy, labor, and delivery with uterine leiomyomas: a population-based study. Obstetrics and gynecology 2000, 95(5):764–769. 10775744 10.1016/s0029-7844(99)00605-5

[3] Laughlin SK, Baird DD, Savitz DA, Herring AH, Hartmann KE: Prevalence of uterine leiomyomas in the first trimester of pregnancy: an ultrasound-screening study. Obstetrics and gynecology 2009, 113(3):630–635. doi: 10.1097/AOG.0b013e318197bbaf 19300327 10.1097/AOG.0b013e318197bbaf PMC3384531

[4] Drayer SM, Catherino WH: Prevalence, morbidity, and current medical management of uterine leiomyomas. International journal of gynaecology and obstetrics: the official organ of the International Federation of Gynaecology and Obstetrics 2015, 131(2):117–122. 10.1016/j.ijgo.2015.04.051 26275638

[5] Donnez J, Hudecek R, Donnez O, Matule D, Arhendt HJ, Zatik J, et al: Efficacy and safety of repeated use of ulipristal acetate in uterine fibroids. Fertility and sterility 2015, 103(2):519–527.e513. doi: 10.1016/j.fertnstert.2014.10.038 25542821 10.1016/j.fertnstert.2014.10.038

[6] Wu JL, Segars JH: Is vitamin D the answer for prevention of uterine fibroids? Fertility and sterility 2015, 104(3):559–560. doi: 10.1016/j.fertnstert.2015.06.034 26187299 10.1016/j.fertnstert.2015.06.034

[7] Donnez J, Vazquez F, Tomaszewski J, Nouri K, Bouchard P, Fauser BC, et al: Long-term treatment of uterine fibroids with ulipristal acetate. Fertility and sterility 2014, 101(6):1565-1573.e1561–1518. 24630081 10.1016/j.fertnstert.2014.02.008

[8] Medikare V, Kandukuri LR, Ananthapur V, Deenadayal M, Nallari P: The genetic bases of uterine fibroids; a review. Journal of reproduction & infertility 2011, 12(3):181–191. 23926501 PMC3719293

[9] Islam MS, Greco S, Janjusevic M, Ciavattini A, Giannubilo SR, D'Adderio A, et al: Growth factors and pathogenesis. Best practice & research Clinical obstetrics & gynaecology 2016, 34:25–36. 26527305 10.1016/j.bpobgyn.2015.08.018

[10] Qidwai GI, Caughey AB, Jacoby AF: Obstetric outcomes in women with sonographically identified uterine leiomyomata. Obstetrics and gynecology 2006, 107(2 Pt 1):376–382. doi: 10.1097/01.AOG.0000196806.25897.7c 16449127 10.1097/01.AOG.0000196806.25897.7c

[11] Ezzedine D N E: Are Women With Uterine Fibroids at Increased Risk for Adverse Pregnancy Outcome? Clin Obstet Gynecol 2016, 59(1):119–127 2016 Mar;59(1):119–27. doi: 10.1097/GRF.0000000000000169 26670833 10.1097/GRF.0000000000000169

[12] Ciavattini A, Clemente N, Delli Carpini G, Di Giuseppe J, Giannubilo SR, Tranquilli AL: Number and size of uterine fibroids and obstetric outcomes. The journal of maternal-fetal & neonatal medicine: the official journal of the European Association of Perinatal Medicine, the Federation of Asia and Oceania Perinatal Societies, the International Society of Perinatal Obstet 2015, 28(4):484–488. 10.3109/14767058.2014.921675 24803127

[13] Vergani P, Locatelli A, Ghidini A, Andreani M, Sala F, Pezzullo JC: Large uterine leiomyomata and risk of cesarean delivery. Obstetrics and gynecology 2007, 109(2 Pt 1):410–414. doi: 10.1097/01.AOG.0000250470.78700.f0 17267843 10.1097/01.AOG.0000250470.78700.f0

[14] Lai J, Caughey AB, Qidwai GI, Jacoby AF: Neonatal outcomes in women with sonographically identified uterine leiomyomata. The journal of maternal-fetal & neonatal medicine: the official journal of the European Association of Perinatal Medicine, the Federation of Asia and Oceania Perinatal Societies, the International Society of Perinatal Obstet 2012, 25(6):710–713. 10.3109/14767058.2011.572205 22409539

[15] Shavell VI, Thakur M, Sawant A, Kruger ML, Jones TB, Singh M, et al: Adverse obstetric outcomes associated with sonographically identified large uterine fibroids. Fertility and sterility 2012, 97(1):107–110. doi: 10.1016/j.fertnstert.2011.10.009 22100166 10.1016/j.fertnstert.2011.10.009

[16] Lam SJ, Best S, Kumar S: The impact of fibroid characteristics on pregnancy outcome. American journal of obstetrics and gynecology 2014, 211(4):395 e391–395. 24705132 10.1016/j.ajog.2014.03.066

[17] Chen YH, Lin HC, Chen SF, Lin HC: Increased risk of preterm births among women with uterine leiomyoma: a nationwide population-based study. Human Reproduction 2009, 24(12):3049–3056. doi: 10.1093/humrep/dep320 19740897 10.1093/humrep/dep320

[18] Stout MJ, Odibo AO, Graseck AS, Macones GA, Crane JP, Cahill AG: Leiomyomas at routine second-trimester ultrasound examination and adverse obstetric outcomes. Obstetrics and gynecology 2010, 116(5):1056–1063. doi: 10.1097/AOG.0b013e3181f7496d 20966689 10.1097/AOG.0b013e3181f7496d

[19] Sheiner E, Bashiri A, Levy A, Hershkovitz R, Katz M, Mazor M: Obstetric characteristics and perinatal outcome of pregnancies with uterine leiomyomas. The Journal of reproductive medicine 2004, 49(3):182–186. 15098887

[20] Noor S, Fawwad A, Sultana R, Bashir R, Qurat ul a, Jalil H, et al: Pregnancy with fibroids and its and its obstetric complication. Journal of Ayub Medical College, Abbottabad: JAMC 2009, 21(4):37–40. 21067021

[21] Klatsky PC, Tran ND, Caughey AB, Fujimoto VY: Fibroids and reproductive outcomes: a systematic literature review from conception to delivery. American journal of obstetrics and gynecology 2008, 198(4):357–366. doi: 10.1016/j.ajog.2007.12.039 18395031 10.1016/j.ajog.2007.12.039

[22] Michels KA, Velez Edwards DR, Baird DD, Savitz DA, Hartmann KE: Uterine leiomyomata and cesarean birth risk: a prospective cohort with standardized imaging. Annals of Epidemiology 2014, 24(2):122–126. doi: 10.1016/j.annepidem.2013.10.017 24321612 10.1016/j.annepidem.2013.10.017 PMC3926444

[23] Morgan Ortiz F, Pina Romero B, Elorriaga Garcia E, Baez Barraza J, Quevedo Castro E, Peraza Garay Fde J: [Uterine leiomyomas during pregnancy and its impact on obstetric outcome]. Ginecologia y obstetricia de Mexico 2011, 79(8):467–473. 21966843

[24] Rosati P, Exacoustos C, Mancuso S: Longitudinal evaluation of uterine myoma growth during pregnancy. A sonographic study. Journal of ultrasound in medicine: official journal of the American Institute of Ultrasound in Medicine 1992, 11(10):511–515. 1404579 10.7863/jum.1992.11.10.511

[25] Moise KJ, Jr.: Ultrasound diagnosis of uterine myomas and complications in pregnancy. Obstetrics and gynecology 1993, 82(5):881–882. 8414345

[26] Febo G, Tessarolo M, Leo L, Arduino S, Wierdis T, Lanza L: Surgical management of leiomyomata in pregnancy. Clinical and experimental obstetrics & gynecology 1997, 24(2):76–78. 9342467

[27] Parazzini F, Tozzi L, Bianchi S: Pregnancy outcome and uterine fibroids. Best practice & research Clinical obstetrics & gynaecology 2016, 34:74–84. 26723475 10.1016/j.bpobgyn.2015.11.017

[28] Kramer MS, Berg C, Abenhaim H, Dahhou M, Rouleau J, Mehrabadi A, Joseph KS: Incidence, risk factors, and temporal trends in severe postpartum hemorrhage. American journal of obstetrics and gynecology 2013, 209(5):449.e441–447. 23871950 10.1016/j.ajog.2013.07.007

[29] Navid S, Arshad S, Qurat ul A, Meo RA: Impact of leiomyoma in pregnancy. Journal of Ayub Medical College, Abbottabad: JAMC 2012, 24(1):90–92. 23855105

[30] Roberts WE, Fulp KS, Morrison JC, Martin JN, Jr.: The impact of leiomyomas on pregnancy. The Australian & New Zealand journal of obstetrics & gynaecology 1999, 39(1):43–47. 10099748 10.1111/j.1479-828x.1999.tb03442.x

